# “Seeds tyranny: No flowers for old plants”

**DOI:** 10.1093/plphys/kiaf201

**Published:** 2025-05-23

**Authors:** María Flores-Tornero

**Affiliations:** Assistant Features Editor, Plant Physiology, American Society of Plant Biologists; Departament de Biologia Vegetal, Facultat de Ciències Biològiques, Universitat de València, Burjassot, Valencia 46100, Spain

Flowering initiation is a crucial step in plant reproduction that has been widely studied, but the end of this process and its regulation remain elusive. End of flowering implies stopping the production of new flowers by arresting the proliferation of inflorescence meristems.

Early pioneering works in tomato (Murneek 1926), pea (Lockhart and Gottschall 1961), and rice (Biswas and Choudhuri 1980) shed some light on this topic by suggesting that fruit development is somehow involved in this proliferative arrest. One of the main working hypotheses considered fruits as a sink of resources that the plant prioritized over the activation of inflorescence meristems, triggering their proliferative arrest (Gifford and Evans 1981; Ho 1996).

Hensel et al. (1994), working with Arabidopsis, observed that new flowers continue to be produced when fruits are not set, demonstrating a connection between meristem proliferation and fruit set. This result clearly suggested the existence of a long-distance signal that induces the proliferative arrest from fruits to inflorescence meristems. More specifically, some authors suggested not the fruits but the seeds as the source of a mobile signal that triggers the end of flowering; hence the name of “death hormone’ (Engvild 1989).

Our current knowledge on flowering termination is built on Arabidopsis, but do similar controls also occur in crops? In this issue of *Plant Physiology*, López-Martín et al. (2025) set out to characterize the end of flowering in *Solanum lycopersicum* var. Micro-Tom. The authors explored the quantitative effect of fruit removal on delaying proliferative arrest by removing none (control), half, or all flowers present in wild-type (WT) plants (Figure). They observed that in plants with half of the flowers, proliferative arrest arrived 5 weeks later than control, while plants with all flowers removed never stopped producing new flowers. Even more interesting is the fact that both control and plants with half of the flowers removed produced approximately the same number of fruits and seeds before reaching the proliferative arrest. Moreover, the authors found that proliferative arrest can be rescued by fruit removal, starting again to produce new flowers and fruits. This indicates that fruits are blocking the development of auxiliary meristems by producing a long-distance signal (Figure).

**Figure.**
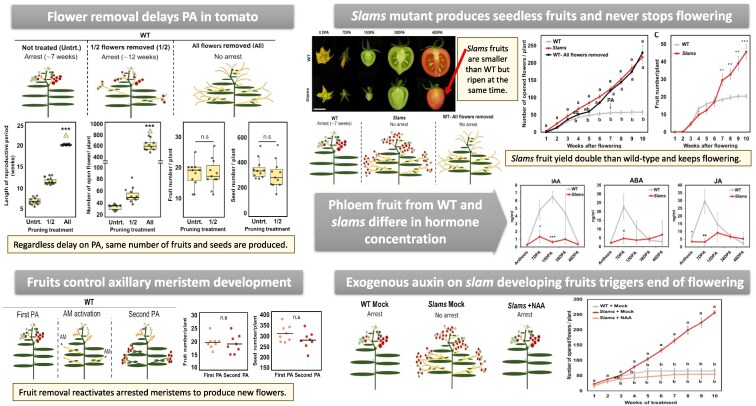
Summary diagram of the experimental flow using Micro-Tom. AM, axillary meristem; PA, proliferative arrest. WT, wild-type. Figures were taken and adapted from López-Martín et al. (2025).

Given that fruits contain seeds, the team wanted to narrow the circle by exploring the role of seeds inside the fruits as the source of this hypothetical signal. To this end, they created the *slams* mutant, a male-sterile tomato plant that produces 100% parthenocarpic (seedless) fruits. In comparison to WT, *slams* tomatoes are smaller, but the ripening time is the same (Figure). Taking advantage of this mutant, the authors compared flower and fruit production between WT, *slams* mutant, and WT with all the flowers removed. Surprisingly, *slams* never stopped flowering, and by the end of the experiment it had doubled the production of fruits yielded by WT without signs of flowering termination (Figure). This interesting experiment supports the seed-derived “death hormone” hypothesis also in tomato, and it prompted López-Martin and collaborators to explore the nature of this signal.

As auxin was known to be involved in flower arrest in Arabidopsis (Walker et al. 2023), the authors decided to analyze phytohormone content in fruit phloem exudates in parthenocarpic and WT plants at different time points, from anthesis until mature fruits (Figure). The team detected significant peaks of auxin, abscisic acid, and jasmonate hormones at early stages of fruit development in WT plants, whereas these phytohormones remained very low and stable in *slams* mutants over time points. This result backs the role of seeds in producing those phytohormones but not in triggering proliferative arrest of inflorescence meristems.

Finally, to establish a link between auxin and proliferative arrest, the authors applied synthetic auxin 1-naphthaleneacetic acid exogenously to *slams* fruits at early developmental stages (Figure). Interestingly, *slams* plants showed a proliferative arrested at similar time as WT plants while producing the same small fruits. This experiment confirms the role of seed-produced auxins as main signals in the pathway of flowering termination.

In general, this study provides evidence on the essential and systemic role of seeds in triggering proliferative arrest by producing a long-distance signal that involves auxin and abscisic acid in the signaling pathway. The elucidation of the remaining components of the signaling pathway represents one of the most exciting questions to address in the near future. Thus, the identification of new players involved in the end of flowering will improve not only our understanding of the process but also our capacity to manipulate it in our favor.

Taken together, the work of López-Martín et al. (2025) fills an essential piece in elucidating the mechanism controlling the end of flowering, holding the biotechnological promise of increasing crop yield by prolonging the flowering time. Every beginning has an end, but not necessarily.

## Data Availability

The data underlying this article are available at https://doi.org/10.1093/plphys/kiaf195.
